# Socio-Emotional Development of Children with Cochlear Implant: A Systematic Review

**DOI:** 10.21315/mjms2021.28.5.2

**Published:** 2021-10-26

**Authors:** Geh Cha Long, Cila Umat, Normah Che Din

**Affiliations:** Centre for Rehabilitation & Special Needs Studies, Faculty of Health Science, Universiti Kebangsaan Malaysia, Kuala Lumpur, Malaysia

**Keywords:** social, emotional, development, children, cochlear implants

## Abstract

**Background:**

Attaining socio-emotional competence is challenging for children with hearing impairment. There is wide recognition of children with cochlear implant (CI) indicating significant improvement in their speech and language abilities, however many factors may restrict their chance of having reciprocal social interactions. A significant improvement in speech and language does not automatically affirm the quality of social interactions. This present observation on social-emotional development addressed a more current representative population of children with hearing loss who have benefitted from cochlear implantation.

**Methods:**

The research conducted a systematic review of selected articles from Scopus and PubMed databases, retrieved through three search-process keywords, namely socio-emotional, children and CI. The inclusion criteria only included journal articles published in English with empirical data from the year 2010–2019. The initial search had identified 189 potential abstracts and after removal of duplicates, only 38 eligible studies met the inclusion criteria.

**Results:**

Among 38 studies reviewed, 19 studies showed comparable socio-emotional skills with peers in social interaction, empathy, emotion theory of mind and comprehension skills. Conversely, the other 19 studies presented underprivileged results in socio-emotional functioning mainly in identifying facial expression, regulating emotion and emotional cues in the auditory domain.

**Conclusion:**

This review concluded that the socio-emotional development among children with CI, both at preschool-age and school-age, was not justified due to the heterogeneity in studies across measurement and small sample size. Also, the conclusion recommended extensive cross-referencing, mixed-mode research design, detailed distinguishing of socio-emotional functioning and identification of diverse groups of the population with impaired hearing as an approach to provide empirical evidence on socio-emotional functioning among children with CI in the future.

## Introduction

In childhood curriculum among those with severe to profound hearing-impairment and children with cochlear implant (CI), certain key areas focus particularly on the auditory development such as hearing, sound, speech and language abilities (1–4). However, it is evident that other micro areas, such as social and emotional, do not get the same emphasis. Intervention among children with CI should not only be limited to auditory and language development, in that it should also focus on socio-emotional development to achieve holistic child development (5). The broadly reported socio-emotional development plays a crucial role in predicting different areas, such as mental health, academic achievement and even job performance (6–7). Such development also hinges on an individual’s self-esteem, the capability to form relationships and empathy skills (8).

Accomplishing socio-emotional competence is challenging for children with impaired hearing as seen in the prevalence of socio-emotional difficulties in them that may range from 8% (9)–41.3% (10). Nowadays, the population size of children with hearing loss shows a decline due to early detection and identification of hearing loss among infants with hearing problems (11). Some of these children who went through early detection had the opportunity to access sound with the help of CI and digital amplification systems and therefore showed significant improvement in their speech and language abilities as compared to those without CI (12–16). Several studies suggested that the outcomes in terms of language abilities were compatible among children with CI and their peers (11–12).

Theoretically, given the positive result in speech and language development among children with CI, the hypothesis indicates a boost in socio-emotional abilities after cochlear implantation (17). The past studies noted the expectation for these children to be able to overcome many of the socio-emotional problems. Unfortunately, reports indicated varying results among children with CI despite the oral language was a predominant mode of social communication and peer interaction. Logically, children with immature language ability may face difficulties in making friends within a mainstream setting (18). Nevertheless, the results suggested that children with CI who have comparable language abilities with their peers were persistently challenged by the social settings as they, too, encountered problems in establishing friendships (19). Such observations were evident in the children’s population in Malaysia. Since 2015, parents have relatively expressed concerns about the below-average level of socio-emotional school readiness skills among children with CI compared to children with normal hearing at the centre for cochlear implantations established in Universiti Kebangsaan Malaysia (UKM) (20–21).

Limited research is available in identifying the factors that may contribute to social incompetence among children with CI. Previous studies reported that a poor listening environment or the inability to differentiate an auditory signal may minimise the chances for reciprocal interactions. Other factors such as inappropriate pragmatic skills practised, immature perception of emotion and lack of strategies to remain in a group or constant failure to initiate communication may exacerbate their development of social abilities (22–25). Thus, the significant improvement of speech and language does not automatically project the good quality of social interaction.

The post-CI stage is a transition phase marked by major psychological and cognitive changes of emerging adulthood. Socio-emotional development among children with CI, neither in preschool- nor school-aged, was yet to be correlated with any specific causal reasons to poor social interaction. This study, therefore, systematically reviewed socio-emotional development concerning children with CI from the year 2010 onwards to address a more current representative population of children with hearing loss who have benefitted from cochlear implantation.

## Methods

The study employed a systematic review that complies with the PRISMA (preferred reporting items for systematic reviews and meta-analyses) guidelines to define clear objectives and permit a rigorous search of terms interrelated to socio-emotional development among children with CI (26).

### Resources

An electronic search was executed on various forms of socio-emotional skills among children with CI by covering relevant literature databases (i.e. Scopus and PubMed). Scopus is one of the most robust databases of peer-reviewed literature with more than 22,800 journals covering various subject areas such as health professions, medicine, psychology and social science. PubMed comprises more than 29 million citations for literature from MEDLINE, NLM’s database of citations and abstracts in the fields of, among others, medicine, healthcare systems and preclinical sciences. All articles in the PubMed database are tagged with organised words on content and main topics to help accessor obtain better search results.

## Systematic Review Process

The study performed a systematic review process in March 2019 involving four stages, namely literature identification, screening, selection of eligible articles and data extraction from the selected articles.[Fig f1-02mjms2805_ra]

### Identification

The study performed a systematic search in Scopus and PubMed using keywords similar and related to social, emotion, children and CI based on thesaurus and past studies (Table 1).

### Screening

Table 2 highlights the eligibility and exclusion criteria for the studies reviewed. Selectively, the screening process only included journal articles published in English from the year 2010–2019 with empirical data to avoid any unnecessary confusions and difficulties in translation. The screening process also excluded review articles, book series, books, chapters in books and conference proceedings. All duplicates of articles were removed during screening. The identified period of nine years (from 2010–2019) worked as a timeline sufficient for the growth of research and related publications.

### Selection of Eligible Articles

After exclusion of articles from screening and removal of duplicates, the targeted articles were assessed and analysed. Specific studies that answered to the formulated questions were then verified through understanding the abstracts and reading the full articles.

### Data Extraction

Two investigators independently screened all unique article titles and abstracts to identify related articles. Disagreements were resolved by consensus. Thirty-eight eligible studies met the inclusion criteria. Data were extracted by reviewer using a pre-piloted form which was developed to list study characteristics including: measurement, research design, sample, findings and limitations. Data were then analysed using thematic method to identify the social and emotional functioning of children with CI.

## Results

Overall, the investigators managed to systematically review 38 studies published on the socio-emotional development of children with CI. All of the studies were quantitative in nature, despite the smaller sample size used. From the total of 38 studies, the majority of the study design, with a total number of 17, was reported to be survey whilst seven were cross-sectional type and 10 were the longitudinal type. Other non-experiment research designs reported included seven correlational studies and 11 causal-comparative studies. Lastly, three studies provided a quasi-experiment research design with matching post-test—only control group type to support this review.

Study populations ranged from 2 months old to 18 years old participants. Ten studies included both preschool- and school-aged children (32, 37–38, 44, 48–49, 53, 56–57, 62). Twenty-eight studies explicitly included either preschoolers (5, 27–29, 30–31, 33–36, 39–40) or school-aged children (38, 41–43, 46–47, 50–52, 54–55, 58–61, 63). For the other six studies, the age range was not reported, however mean age was provided frequently (5, 27, 31, 42, 52, 56). Six studies provided outcomes of social competence only (28, 30, 36, 44, 53, 59), 13 studies provided emotional functioning only (5, 31, 35, 37, 42–43, 48, 52, 54–55, 58, 61, 63), whilst the remaining 19 studies provided a combination of social competence and emotional functioning for pre-school children (27, 29, 32–34, 38–40) and school-aged children (38, 41, 46–47, 49–51, 56–57, 60, 62). Tables 3 and 4 included a comprehensive summary of studies including the authors, measures, research designs, samples, findings and limitations of the described socio-emotional development among children with CI.

### Socio-Emotional Development Among Preschool-Aged Children with Cochlear Implant

For preschool-aged children with CI, the study identified and reviewed a total of 15 studies (Table 3). Eight studies reported that children with CI showed positive social and emotional skills with peers (27–34). Whilst findings from six studies among young preschoolers with an average age of 3 years old reported that these children were able to demonstrate equivalent skills of social interaction (27–30, 33–34) and empathy (28, 30) in the survey, causal-comparative and correlational studies. The remaining two studies among preschoolers with an average age of 6 years old observed comparable social competency in older preschool-aged children with CI evident in their choice of recognising, processing and producing emotional content relevant to their environment (31–32). The combination of early implantation and a regular auditory-oral-based therapy both played a crucial part in the positive outcomes for social and emotional functioning reported.

In contrast to the eight studies that reported positive social and emotional skills with peers in the children with CI, samples from the remaining seven studies reported underprivileged results of socio-emotional functioning among preschoolers with CI compared to their peers (5, 35–40). In these studies, preschoolers’ ages ranged 2 years old–6 years old and their social skills impairment were studied using the quasi-experiment, survey, causal-comparative and correlational studies. The standardised developmental (Child development inventory [CDI]; INSITE developmental scale) (36, 39–40) and behavioural tests (Strengths and difficulties questionnaire [SDQ]; Behaviour assessment system for children-2nd edition [BASC-2]) (36, 38–39) were administered to determine the poor socio-emotional development among these children with CI. Delayed facial expression identification (5, 37), poor emotional regulation strategies (36) and the limited ability to understand conveyed messages (40) contributed to low social competency among these preschoolers with CI.

### Socio-Emotional Development Among School-Aged Children with CI

A total of 23 studies were reviewed for socio-emotional development among school-aged children with CI (Table 4). Eleven studies showed a diminutive difference in the level of social and emotional skills between children with CI and those with normal hearing (41–51). Children with CI with an average younger school-aged 6 years old–9 years old were observed to present compatible emotion theory of mind and comprehension skills (48, 52) compared to their peers (53). Whilst the result informed that older school-aged children with CI (10 years old–12 years old) were less likely to experience difficulties in emotion (42, 46). These children were able to show voice emotion recognition comparable to adults with normal hearing (43).

On the contrary, 12 studies testified below the age-appropriateness level of socio-emotional development among school-aged children with CI. Certain studies reported that, although some CIs were implanted at an earlier age, school-aged CI children still persistently showed difficulties in socio-emotional functioning irrespective of their age groups (38, 53–54, 61–63). Moreover, some results outlined that the socio-emotional development of children with CI was significant in a clinical range, although CI were implanted since below the age of 4 years old (60). A few studies reported that children with CI had poor emotional perception (58, 61) and were less accurate in identifying emotion cues in the auditory domain (54, 63). They also presented a lack of empathy and lack of prosocial motivation to interact with peers (59).

## Discussion

From the review, there were only two studies that integrated child, caregiver and teacher’s responses (50, 56). Such limitation restricted the holistic view for researchers who attempted to create a triangulated profile of the socio-emotional development of children with CI. Triangulated profile is a two-pronged approach that can provide better results of socio-emotional skills and reduce bias with its numerous perspectives from different groups of the respondent. Indeed, one must factor in the time consumption and cost in collecting data from diverse groups, especially given the typically low-response rates of young children due to their immaturity in verbal expression. Furthermore, researchers may take a longer time to observe and interpret children’s behaviours or performance. However, following the relatively low concurrence between child, caregiver and teacher’s responses in this area, child’s task performance and caregiver’s view are still the priority in measuring the socio-emotional development among children with CI.

It is worth noting that there were different measures used to determine the socio-emotional development of children with CI, creating difficulties for researchers to conclude this area of research. Based on the systematically reviewed studies among preschool- and school-aged children with CI, the results reported almost equal distribution, neither average nor poor socio-emotional development, compared to peers. The focus area of every research for child’s task performance may be different, varying wide-range areas of empathy and social interaction (59), facial expression recognition (5, 37), and emotional identification and regulation (36, 54, 58, 61, 63) among others. Meanwhile, a variety of caregiver-based questionnaires may report different socio-emotional outcomes of children due to the non-standardised scales of each questionnaire. For example, SDQ emphasised the domains of emotional symptoms, conducted problems, hyperactivity, peer-relationship problems and prosocial behaviour (30, 36, 39, 50, 56–57) whilst BASC–2 showed the domains of externalising problems, internalising problems, behavioural symptoms index, and adaptive skills (38). The result also suggested that an effective intervention plan included developing a practical, standardised measurement to capture certain socio-emotional domains of children with CI.

Another observation from this review denotes the imbalance among the types of data collection. Many more data collections tapped into the quantitative, as opposed to the qualitative or the mixed-mode method. The majority of reviewed studies indicated a preference for reports on quantitative data, although numbers of respondents were mostly small due to the unique population of cochlear implantation. On the contrary, qualitative research is almost always the starting point to discover new problems of a special population. Researchers might have overlooked certain areas of socio-emotional development as there was a myriad of studies designed to focus on quantitative data collection. Thus, it is advisable to find a balance between in-width and in-depth in data analysis to draw a reliable and comprehensive understanding of socio-emotional development among children with CI. More qualitative and/or mixed-mode research are encouraged to justify the components of socio-emotional development feasible to cochlear implantation population.

## Recommendations

This systematic review has thoroughly analysed the 38 selected studies. The studies comprised both preschool- and school-aged children with CI. A clear conclusion on the socio-emotional functioning among children with CI, however, could not be drawn due to the heterogeneity of these selected studies evident in several factors such as age, sample size, and varying designs and instruments. Another explanation for having divergent conclusions is likely due to the insufficient numbers of studies included in this study. The review did not include unpublished studies and the studies published in the non-English medium. Moreover, it did not perform an extensive cross-referencing of the reference lists from the papers retrieved in the electronic database. These methodological limitations could have, therefore, potentially excluded several relevant past studies.

Most of the studies involved were conducted using a small sample size. Reporting convincing results and generalising observations to the population level when sampling was deemed limited was difficult. The population with hearing impairment was considered a special group, therefore, reaching out and recruiting participants from this population were challenging. More studies with a mixed-mode design were highly suggested for the future to circumvent the limitations of small sample size in order to obtain more conclusive and convincing outcomes.

Some studies also indicated that music was one of the optimal stimuli to examine the abilities of children with CI as a way to distinguish emotions (54, 58, 61, 63–64). A capability in identifying emotional content from the auditory stimuli, such as music with variations in pitch, amplitude and timing characters, may play an essential role in the lives of these children. CI provides the opportunity to restore not only sound but also the access to emotional cues through hearing. However, children with CI with good ability in distinguishing music emotions may not be able to anticipate quality social interaction. The lack of human voice stimuli may still potentially affect these children’s ability in vocal emotion identification and may indirectly interfere with their socio-emotional functioning.

Majority of the studies that underwent systematic review here were reliant on caregivers for the questionnaires (i.e. SDQ and Empathy questionnaire) assessing socio-emotional functioning of children with CI (33, 35–36, 38–39, 50–51, 57, 60, 62). These questionnaires were short, easy to be administered and useful for caregivers to screen the children’s level of socio-emotional development. However, these questionnaires did not measure socio-emotional extensively. The child would need to be immediately referred to mental health professionals for a more in-depth and precise examination of overall development functioning. It is uncertain how the absence of more clinically sensitive methods may affect the level of socio-emotional development in children with CI.

## Conclusion

This systematic review highlighted the trend of research on socio-emotional development among children with CI. Overall outcomes of these research were varied. As discussed, a limited number of studies and heterogeneity factors (i.e. age, sample size, design and instruments) may have caused the failure to draw a considerable conclusion in this review. Many of the measure in the studies reviewed did not precisely distinguish between different categories and degree of social and emotional abilities. Furthermore, the bias in results interpretation was likely to happen as most data collection was not based on a triangulated profile, which mostly only included the child and the caregiver’s responses. Health professionals for children with CI and mental health researchers should put more effort to encourage more extensive future studies, particularly qualitative and/or mixed-mode methods on socio-emotional development among children with CI. Future studies should take into account of diversities among children with hearing impairment too because that will provide the empirical evidence and the prevalence of socio-emotional development in Malaysia.

## Figures and Tables

**Figure 1 f1-02mjms2805_ra:**
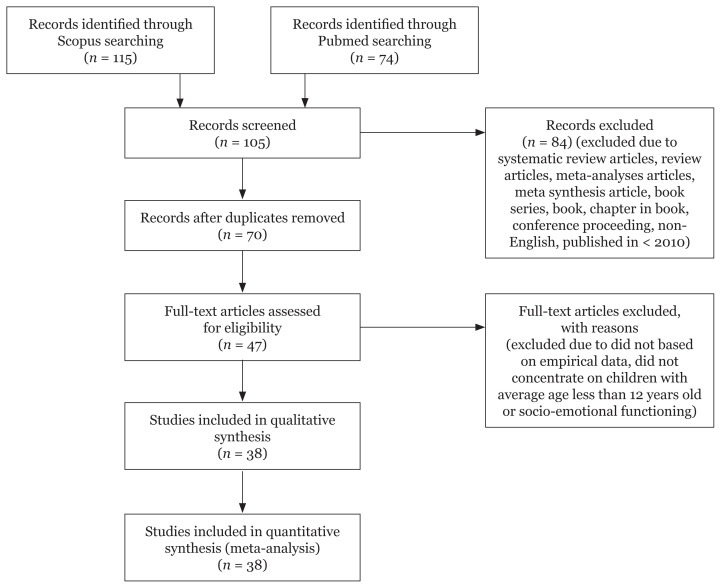
Flow diagram of selection articles

**Table 1:** Keywords and searching strategy

Databases	Keywords used
Scopus	TITLE-ABS-KEY [(social* OR socio*) AND (emotion*) AND (child* OR kid* OR youth* OR preschooler*) AND (cochlear implant*)]
PubMed	[(socio* OR social*) AND (emotion*) AND (child* OR kid* OR youth* OR preschool*)] AND cochlear implant*

**Table 2:** Inclusion and exclusion criteria

Criterion	Eligibility	Exclusion
Literature type	Journal (research articles)	Journals (systematic review), book series, book, chapter in book, conference proceeding
Language	English	Non-English
Timeline	Between 2010 and 2019	< 2010

**Table 3:** Studies investigating the socio-emotional development of preschool-aged children with CI

Author(s)	Measure: assessor	Research design: type	Sample	Main findings	Limitations
Huttunen and Välimaa (27)	Semi-structured questionnaires: parent	Survey: longitudinal	Children with CI (*n* = 18; mean age = 3 years 4 months old)	Socio-emotional of majority children benefited from CI in building self-confidence, sense of safety, expanded social life and positive changes in children’s behaviour	Small sample size
Jackson et al. (35)	Family quality of life (FQoL) scale: caregiver	Survey: cross-sectional	Deaf or hard of hearing children (*n* = 207; mean age = 44 months old; range age = 2 months old–72 months old; *n* with CI = 103; *n* with hearing aid = 95)	Lower satisfaction with emotional well-being among deaf children. Sensory device used by deaf children did not have a significant effect on emotional well-being	Respondents primarily of mothers; Limited populations to be generalised
Ketelaar et al. (28)	Broken car task, copy task, bottle task, empathy task: researcher	Causal-comparative	Children with CI (*n* = 14; mean age = 39 months old; range age = 1 year old–5 years old; mean age of CI activation = 14 months old) versus Children with normal hearing (*n* = 28, mean age = 39 months old)	Children with CI showed empathy and social interactions but were shown less shame compared to hearing peers	Small sample size
Tasker et al. (29)	Naturalistic free-play task: researcher; Four semi-structured joint attention-eliciting tasks: researcher; Caregiver perception scale and adaptive social behavioural inventory: mother	Causal-comparative	Children with CI (*n* = 9; mean age = 29.67 months old; range age = 20 months old–41 months old; mean age at CI implementation = 16.89 months old; duration using CI = 12.22 months old) versus Children with non-CI (*n* = 17; mean age = 24.82 months old; range age = 17 months old–35 months old) versus Children with normal hearing (*n* = 29; mean age = 26.37 months old; range age = 18 months old–36 months old)	Children with CI display more joint attention and were reported as higher on expressive and compliance behaviours and lower on disruptive behaviour which comparable to normal hearing peers. CI may aid in the early socio-emotional development of some deaf children	Short experience with CI; Small sample size; Fail to provide a full developmental picture
Wang et al. (5)	Experiment 1 Facial expression recognition task: researcher Experiment 2 Facial expression recognition task; PPVT-R: researcher	Quasi-experiment: Matching post-test-only control group	Experiment 1 Children with CI (*n* = 5; mean age at CI activation = 23 months old; mean age = 37 months old) and HA (*n* = 11; mean age = 46 months old; mean age at HA fitted = 23 months old) versus Children with normal hearing (*n* = 16; mean age = 43 months old) Experiment 2 Children with CI (*n* = 5; mean age = 56 months old; mean age at CI activation = 22.8 months old) and HA (*n* = 11; mean age = 65 months old; mean age of HA fitted = 31 months old) versus Children with normal hearing (*n* = 16; mean age = 62 months old)	Experiment 1 There was a delay in pre-schoolers with CI or HA on facial expression recognition compared to hearing peers Experiment 2 Early emotional intervention was necessary to help children with CI or HA catch up with normal hearings	Small sample size; Social-cognitive component (e.g. false belief understanding) was not measured among children with CI and HA
Wiefferink et al. (36)	CDI; SDQ; Empathy questionnaire: parent Emotion regulation task; Coping task: researcher	Causal-comparative	Children with CI (*n* = 69; mean age = 41 months old; range age = 1.5 years old–5 years old; mean age at CI activation = 19 months old) versus Children with normal hearing (*n* = 67; mean age = 44 months old; range age = 1.5 years old–5 years old)	Children with CI had fewer emotion regulation strategies and were less socially competent than peers. They expressed negative emotions more often and more intensely	CI children at this young age are not yet evident in externalising problems
Ketelaar et al. (30)	Empathy questionnaire; Emotion expression questionnaire; SDQ: parent	Survey: longitudinal	Children with CI (*n* = 61; mean age = 39 months old; range age = 14 months old–77 months old; mean age at CI activation = 16 months old;) Children with normal hearing (*n* = 89; mean age = 39 months old; range age = 14 months old–77 months old)	Children with CI showed equal levels of social competence or empathic behaviour compared to peers	It is unclear whether frequent eye contact by children with CI that implies more attention to the emotional impact or visual cues
Ziv et al. (31)	Language test PPVT-R: researcher Emotion understanding test Emotion identification from faces; Understanding emotions elicited in a typical context; Affective perspective taking from stories: researcher Theory of mind test Understanding of false belief: researcher	Causal-comparative	Children with CI (*n* = 20; mean age = 6.6 years old; mean age at CI implantation = 2.5 years old) and deaf signers (*n* = 10; mean age = 6.2 years old) versus Children with normal hearing (*n* = 23; mean age = 5.1 years old)	Children with CI and normal hearing showed comparable performance results in understanding emotions in typical contexts and false belief	Limited information about the participants’ linguistic skills
Mildner and Koska (32)	Perception test 1: Each child’s name was pronounced in combination with the five emotions: researcher Perception test 2: Nonsense-syllable word pairs with the emphasise on the first syllables were combined with the five emotions: researcher	Causal-comparative	Children with CI (*n* = 3; range age = 6–7 years old; age at CI implantation = 1.08 years old) versus Children with normal hearing (*n* = 3; range age = 6 years old–7 years old) versus Children with normal hearing (*n* = 3; range age = 4 years old–5 years old)	Children with CI and normal hearing showed comparable result in processing and producing emotional content. Early implantation and regular auditory-oral based therapy is crucial	Data set is too small
Ketelaar et al. (33)	Broken car task, copy task and bottle task, cooperation scale; Reynell developmental language scales: researcher SDQ; Emotion vocabulary questionnaire: parent	Correlational	Children with CI (*n* = 60; mean age = 38 months old; range age = 14 months old–61 months old; mean age at CI implantation = 16 months old) Children with normal hearing (*n* = 184; mean age = 38 months old; range age = 14 months old–61 months old)	Children with CI expressed moral emotion to a lesser extent and seemed to be less aware compared to peers. However, they demonstrated comparable social skills	Preliminary study; Biased from parents’ report
Wang et al. (37)	Colour images of basic emotions (happiness, sadness, anger and fear); Black and white images of four shapes (circle, square, rectangle, triangle): researcher	Quasi-experiment: Matching post-test-only control group	Children with CI (*n* = 10; mean age = 54.86 months old; range age = 30 months old–84 months old; period of CI activation = majority used over half a year) versus Children with normal hearing (*n* = 22; mean age = 54.41 months old)	Children with CI were reported significant developmentally delayed in their emotion-labelling (verbal) and -matching (nonverbal) tasks compared to peers	Small sample size; Rehabilitation period for children with CI were relatively short; Stimuli images were adults; Wide range age of children with CI
Freeman et al. (38)	Beginner’s intelligibility test: researcher Behaviour assessment system for children-2nd edition: parent	Survey: longitudinal	Children with CI (*n* = 27; mean age = 2.8 years old; range age = 6 months old–5 years old; age of CI implantation < 3 years old) Children with normal hearing (*n* = 30; range age = 3 years old–6 years 11 months old)	Children with CI performed significantly more poorly on psychosocial behaviour	Non-random sampling; Small sample size; bias parent report
Ketelaar et al. (34)	Negative emotion expression scale; Empathy questionnaire; Parenting style; Comprehension and expression scales: parent Emotion-regulation task; Reynell developmental language scales; Schlichting expressive language test: researcher	Correlational	Parent and children with CI (*n* = 46; mean age = 37.7 months old; range age = 14 months old–61 months old; mean age of CI = 16 months old) Parent and children with normal hearing (*n* = 46; mean age = 37.6 months old; range age = 15 months old–61 months old)	Group of children with CI most often showed positive social-emotional functioning. Hearing status did not moderate relationship between parenting styles and children’s social-emotional functioning	Not able to make firm statement; Validity and reliability of the several instruments; Did not determine sign-language proficiency of parents; Heterogenous group of children with CI
Wong et al. (39)	SDQ; CDI; Functional auditory behaviour: parent Wechsler non-verbal scale of ability; Preschool language scale-fourth edition: researcher	Survey: longitudinal	Children with CI (*n* = 120; mean age = 61.6 months old; range age = 58 months old–73 months old; mean age of CI activation = 16.27 months old) Children with HA (*n* = 236; mean age = 61.6 months old; range age = 58 months old–73 months old; mean age of HA fitted = 10.68 months old)	Children with CI and HA have clinically significant psychosocial problems in emotional and social even though they develop good language ability	Heterogeneity of hearing impairment group; Reliance on parent report; Amount of missing data; Lack of variables contribute to psychosocial outcomes
Pilarska and Sekula (40)	INSITE developmental scale: researcher State-trait anxiety inventory (STAI): mother	Correlational	Children with CI (*n* = 21; mean age = 19.52 months old; range age = 13 months old–24 months old; range age of CI implantation = 12 months old–18 months old) Children with HA (*n* = 23; mean age = 19.09 months old; range age = 12 months old–24 months old) Children with normal hearing (*n* = 27; mean age = 19.11 months old; range age =13 months old–24 months old)	Children with CI were reported to have poorer social and emotional development than children with normal hearing. It may cause by the limited ability to understand messages from surroundings	Small sample size

Note: HA = Hearing-aid

**Table 4:** Studies investigating the socio-emotional development of school-aged children with CI

Studies	Measure: assessor	Research design: type	Sample	Main findings	Limitations
Loy et al. (41)	KINDL-R questionnaire for measuring health-related quality of life: parent and child	Survey: Cross-sectional	Group 1 Children with CI (*n* = 52; mean age = 9.1 years old; range age = 8 years old–11 years old; mean age at CI implementation = 3.37 years old; duration using CI = 5.71 years old) Group 2 Children with CI (*n* = 34; mean age = 13.7 years old; range age = 12 years old–16 years old; mean age at CI implementation = 5.83 years old; duration using CI = 7.81 years old)	Children with CI in both groups generally report positive QoL scores in emotional and social well-being which similarly to normal hearing peers and parents.	Generic health-related QoL questionnaire; No data related to levels of speech and language development
Hopyan et al. (54)	Thirty-two brief musical excerpts by judging happy/sad or by pointing smile/frown face: researcher	Quasi-experiment: Matching post-test-only control group	Children with unilateral CI (*n* = 18; range age = 7 years old–13 years old; mean age at CI activation = 2.9 years old) versus Children with normal hearing (*n* = 18; mean age = 10 years 3 months old, range age = 8.08 years old–13.67 years old)	Children with CI were able to distinguish faces emotion in music but do less accurately than hearing peers. They had difficulties identifying emotional cues in the auditory domain	Children with CI may only rely on music rhythm cues to discriminate emotions as sad musical excerpts were generally slower in tempo compared to happy excerpts
Theunissen et al. (55)	CDI; Modified self-report coping scale; Mood questionnaire: child	Causal-comparative	Children with CI (*n* = 27; mean age = 11 years 8 months; range age = 100 months old–192 months old; range age at CI implanted = 11 months old–10y8m) and HA (*n* = 56; mean age = 12 years 3 months; range age = 110 months old–188 months old) versus Children with normal hearing (*n* = 117; mean age = 11 years 8 months old; range age = 114 months old–176 months old)	CI recipients and HA children reported similar levels of depression. Hearing-impaired children were more prone to have poor emotional functioning in depression compared to normal hearing children	Heterogeneity of severity hearing loss among hearing impairment group
Anmyr et al. (56)	SDQ: parent, child, teacher	Causal-comparative	Younger children with CI (*n* = 12; mean age = 9 years old; range age at CI fitted = 0 years old–9 years old)	Children with CI were self-reported to have more difficulties in emotional symptoms and conduct problems compared to parents’ and teachers’ assessment	Small sample size; Majority children had limited language skills
Marschark et al. (53)	Questionnaires tapping children’s and parents’ perceptions of academic functioning and academically related social functioning: parent, child	Causal-comparative	Children with CI (*n* = 15; range age = 5 years old–12 years old; mean age at CI implantation = 33.75 months old)	Children and parents agreed that children with CI were less socially successful than normal hearing peers. CI did not guarantee greater social success	Preliminary analyses due to small sample size
Theunissen et al. (42)	Fear survey schedule for children-revised: child Child symptom inventories-4: parent	Survey: longitudinal	Children with CI (*n* = 32; mean age = 11 years old; mean age at CI activation = 4.6 years old) and HA (*n* = 51; mean age = 12 years old) Children with normal hearing (*n* = 127; mean age = 11 years old)	Children with early received CI demonstrated similar levels of anxiety with and comparable to normally hearing children than to HI children with conventional hearing aids. CI appeared to have a positive influence on the prevention of anxiety, suggesting fewer social obstacles	Heterogeneity of the hearing impairment group
De Giacomo et al. (57)	Leiter International performance scale-revised (Leiter-R); Vineland adaptive behaviour scale (VABS): researcher SDQ: parent	Correlational	Children with CI (*n* = 20, mean age = 9.17 years old; range age = 5 years old–15 years old; mean age at CI implantation = 37.5 months old; mean used device of CI = 79.5 months old) Children with normal hearing (*n* = 20; mean age = 10.08 years old; range age = 4 years old–16 years old)	Children with CI reported to have more difficulties in emotional symptoms and peer problems compared to normal hearing children. Early CI may enhance speech and language development and later decrease emotional problems and improve positive relationships	Small sample size; clinical ascertainment bias due to different population-based study
Chatterjee et al. (43)	Twelve sentences were selected and spoken by one male and one female talker with five emotions (angry, happy, neutral, sad and scare); Listen one presentation of the sentence, and indicated from a closed set which emotion thought was associated with it: researcher	Survey: longitudinal	Children with CI (*n* = 36; mean age = 12.15 years old; range age = 6.83 years old–18.44 years old; mean used device of CI = 8.76 years old) Children with normal hearing (*n* = 31; mean age = 10.76 years old; range age = 6.38 years old–18.76 years old) Adult with normal hearing (*n* = 10; mean age = 23.9 years old) Adult with CI (*n* = 9; mean age = 23.9 years old; range age = 27.34 years old–69.81 years old; mean age = 52.16 years old; mean duration of device use = 8 years old)	Most of the children with CI showed comparable result in the adult normal hearings’ scores with less likely to experience difficulties in voice emotion recognition.	Small sample size; Too limited scope to have a full understanding of the issues underlying the large variability in children with CI
Nasralla et al. (44)	Vineland social maturity scale (VSMS); Columbia mental maturity scale (CMMS); Free drawing activity; Bender visual motor gestalt test and pre-bender visual motor Gestalt test: researcher	Survey: longitudinal	Children with CI (*n* = 20; range age = 1 year 11 months old–13 years old)	Majority of children with CI showed good sociability in giving attention and communication development	Heterogeneity of the hearing impairment group
Shirvani et al. (58)	Raven’s 32-item nonverbal intelligence test; Peretz test: researcher	Survey: Cross-sectional	Children with unilateral CI (*n* = 25; range age = 6 years old–8 years old; mean age at CI activation = 3.6 years old; mean used device of CI = 3.3 years old) Children with normal hearing (*n* = 30; range age = 6 years old–8 years old)	Children with CI had demonstrated poorer emotional perception of music than peers and may negatively affect quality of life and social relationships	Lack of music stimuli; Quality of implant prostheses may affect musical perception
Sundqvist et al. (45)	Sally-Anne procedure; Social-emotional theory of mind (ToM) test (SET): researcher	Correlational	Children with early CI (*n* = 8; mean age = 78.88 months old; mean age at CI implantation = 17.63 months old; mean used device of CI = 61.25 months old) Children with late CI (*n* = 8; mean age = 84.63 months old; mean age at CI implantation = 40.63 months old; mean used device of CI = 44 months old) Children with normal hearing (*n* = 18; mean age = 6.6 years old)	Early CI-group did not differ in emotional ToM compared to normal hearing group. However, late CI-group differed significantly from the comparison group on emotional ToM	Small sample size; Lack data on early interaction experience of deaf children in families; heterogeneous of CI group
Anmyr et al. (46)	Children’s sense of coherence (CSOC) scale; SDQ: child	Survey: cross-sectional	Children with CI (*n* = 19; range age = 9 years old–12 years old; mean age at CI implantation = 4.6 years old; mean used device of CI = 6.2 years old)	Majority of children with CI were reported to have comparable mental health (i.e. less emotional symptoms, less conduct problems, more prosocial behaviour) to normal hearing children	Small sample size
Langereis and Vermeulen (47)	Auditory speech perception abilities; Reynell developmental language scale; CITO; semi-structured interviews on general well-being: researcher	Survey: longitudinal	Children with CI (*n* = 58; range age at CI implantation = 31 months old–35 months old; range age at testing after 60 months old post-implantation = 96 months old–98 months old)	Majority of children with CI showed comparable social-emotional well-being in hard-of-hearing and mainstream education. Meanwhile, parents reported that most children were vulnerable in deaf education	Heterogeneous of CI group
Netten et al. (59)	Empathy questionnaire for children and adolescents (EMQue-CA); Emotion awareness questionnaire (EAQ): child Emotion expression questionnaire (EEQ): parent Observation of empathy; Wechsler intelligence scale for children-third edition (WISC); Clinical Evaluation of language fundamentals-fourth edition (CELF): researcher	Causal-comparative	Children with CI (*n* = 52; mean age = 11.8 years old; range age = 100 months old–194 months old; mean age at CI implantation = 44.5 months old; mean used device of CI = 99 months old) versus Children with HA (*n* = 70; mean age = 12 years old; range age = 110 months old–188 months old; mean used device of HA = 31.2 months old) versus Children with normal hearing (*n* = 162; mean age = 11.9 years old; range age = 99 months old–176 months old)	Children with CI and HA reported lower levels of cognitive empathy and prosocial motivation than normal hearing children, regardless type of hearing device	Small sample size
Mancini et al. (48)	Test of emotion comprehension: researcher	Survey: longitudinal	Children with CI (*n* = 72; mean age = 8.1 years old; range age = 4 years old–11 years old; period of CI activation = > 36 months)	Majority of children with CI were reported to have normal range of emotional comprehension skills; Early implanted with early intervention allowed children to have adequate emotion comprehension and communication development	Small number of children in each age group; Did not investigate other aspects in influencing emotional development; No longitudinal data for maturation process
Park et al. (60)	Categories of auditory performance: researcher Child behavior checklist: parent	Survey: cross-sectional	Children with CI (*n* = 93; age at CI implantation = < 4 years old; used device of CI = > 10 years old)	Relatively large number of the children with CI were being reported that their socio-emotional development scores were in the clinical range	Caregiver’s recall bias; selection bias; only assess 10-year outcomes
Shirvani et al. (61)	Raven’s 32-item nonverbal intelligence test; Persian version of auditory perception test; Peretz’s test: researcher	Causal-comparative	Children with unilateral CI (*n* = 25; mean age = 6.94 years old; range age = 6 years old–8 years old; year of CI activation = > 2 years old) versus Children with bimodal fitting CI (*n* = 20; mean age = 7.07 years old; range age = 6 years old–8 years old; Year of CI activation = 1.65 years old) versus Children with normal hearing (*n* = 30; mean age = 6.84 years old)	Children with unilateral and bimodal fitting CI achieved significant lower score in emotional perception of music test compared to peers. However, children with bimodal fitting performed better than children with unilateral CI	Frequency range of music perception is far beyond the frequency range of children with CI and hearing aids
Freeman et al. (38)	McGarr sentence intelligibility test: researcher Behaviour assessment system for children-2nd edition: parent	Correlational	Children with CI (*n* = 51; range age = 7 years old–20 years old; age of CI implantation < 7 years old) Children with normal hearing (*n* = 47; range age =7 years old–20 years old)	Children with CI performed significantly more poorly on mostly psychosocial behaviours	Non-random sampling; Small sample size; bias parent report
Haukedal et al. (62)	Health-related quality of life (HR-QoL): parent British picture vocabulary test-2nd edition; Clinical evaluation of language fundamental-4th edition: researcher	Causal-comparative	Children with CI (*n* = 106; mean age = 110.07 months old; range age = 60.58 months old–155.73 months old; mean used device of CI = 75.94 months old) versus Children with normal hearing (*n* = 80; mean age = 114.16 months old; range age = 68.11 months old–158.82 months old)	Children with CI were revealed significantly lower scores on the social functioning and psychosocial health than children with normal hearing. However, children with CI demonstrated comparable emotional functioning	Parents’ subjective perception; Only limited specific group of children with CI
Nasralla et al. (49)	Developing questionnaire: parent	Survey: cross-sectional	Children with CI (*n* = 14; mean age = 6 years 5 months old; range age = 2 years 5 months old–13 years 6 months old; mean used device of CI = 3 years 6 months old)	Children with CI showed improvement in socio-emotional abilities particularly reaction to sound which demonstrating name recognition and increase eye contact provided emotional benefits	Parents’ subjective perception about children’s real potential due to acceptance of children’s limitations
Sarant et al. (50)	SDQ: parent Preschool language scale-4; Clinical evaluation of language fundamentals-4; Wechsler non-verbal scale of ability; Wechsler preschool and primary scale of intelligence: researcher Moeller’s family rating scale: teacher	Survey: longitudinal	Children with CI (*n* = 159; range age = 8 years old–17 years old; age of first CI implantation < 3.5 years old; age of second CI implantation < 6 years old)	Children with early CIs were reported to have comparable psychosocial development with peers with normal hearing, with the exclusion of delayed prosocial behaviour development	Samples tend to be much smaller than normative samples
Waaramaa et al. (63)	Listening tests to vocal stimuli for emotion identification (excitement, fear, anger and contentment): researcher	Correlational	Children with CI (*n* = 25; mean age for boy = 11 years old; mean age for girl = 12 years old; range age = 8 years old–17 years old) versus Children with normal hearing (*n* = 18; mean age = 12 years old)	Children with CI identified the emotions with significant less accuracy compared to peers. Acoustic structures of vocal emotional expressions and musical interests may improve children with CI emotional and social development	Difference maturity stage in group of children with CI
Michael et al. (51)	SDQ: parent	Survey: cross-sectional	Children with CI (*n* = 31; range age = 6 years old–19 years old; mean age of CI implantation = 47.23 months old) Children with HA (*n* = 32; range age = 6 years old–19 years old; mean age device HA fitted = 38.56 months old)	Children with CI reported better social-emotional functioning of children with HA. CIs group showed lower levels of hyperactivity/inattention and higher levels of pro-social behaviours.	Perception of parents may be subjective and limited to family setting; Small sample size to do comparison; Only certain social-emotional aspects were measured

Note: HA = Hearing-aid
